# Joint modelling of longitudinal AD cognitive markers and incident MCI/dementia quantifies the longitudinal cognitive—AD risk relationship

**DOI:** 10.1002/alz.091098

**Published:** 2025-01-09

**Authors:** Vahan Aslanyan, Nancy E Ortega, Wendy J Mack, Judy Pa

**Affiliations:** ^1^ Department of Population and Public Health Sciences, Keck School of Medicine, University of Southern California, Los Angeles, CA USA; ^2^ Alzheimer’s Disease Cooperative Study (ADCS), University of California San Diego, La Jolla, CA, USA, La Jolla, CA USA; ^3^ Neurosciences Graduate Program, University of California San Diego, La Jolla, CA, USA, La Jolla, CA USA; ^4^ Alzheimer’s Disease Cooperative Study (ADCS), University of California, San Diego, La Jolla, CA USA

## Abstract

**Background:**

Randomized clinical trials for AD increasingly use composite continuous cognitive endpoints to establish efficacy. While these endpoints provide necessary research and clinical evidence, they do not inform the potential impact on incident MCI/AD. Similarly, studies designed with disease progression as an endpoint fail to incorporate within‐person longitudinal cognitive and functional changes in assessment of disease risk, often relying on baseline measures only, or explore cognitive and functional performance change in separate analyses, independent of conversion. This study incorporates longitudinal change in cognition (measured by Preclinical Alzheimer’s Cognitive Composite (PACC)) and dementia severity (measured by Clinical Dementia Rating—sum of boxes (CDR‐SB)) to measure how longitudinal changes in cognition and function may relate to the risk of disease progression.

**Method:**

Participants were selected from the Harvard Aging Brain Study, a longitudinal study collecting demographic, neuropsychological, and APOE genotyping outcomes. Longitudinal changes in CDR‐SB and PACC score were modelled jointly with conversion to MCI/dementia. All models were adjusted for baseline age, sex, education, and APOE‐e4 carrier status.

**Result:**

287 participants (27 conversions to MCI/dementia, 59.1% women, baseline age = 73.7 years (SD = 6.2), 27.6% APOE‐e4+, education = 15.8 years (SD = 3.09)) collectively had 1511 observations over a median follow‐up of 2.2 years (range 0‐8.7 years).

After adjusting for covariates, PACC score changed by ‐0.016/year (95%CI:(‐0.032, 0.000)) and was associated with hazard ratio (HR) = 1.0405 for progression (95%CI:(1.029,1.054). Survival analysis with baseline PACC score underestimated the association of PACC score and conversion (HR = 1.028 per ‐0.016 between‐individual difference in PACC score, 95% CI: (1.02,1.036)).

Similarly, within‐individual CDR‐SB changed by 0.025/year (95%CI:(0.015,0.035)). This change corresponded to HR = 1.081 for progression (95%CI:(1.053,1.111)). Survival analysis with baseline CDR‐SB underestimated the association of CDR‐SB and rate of conversion to MCI/AD (HR = 1.057 per 0.025 between individual difference CDR‐SB, 95%CI:(1.042,1.073)).

**Conclusion:**

This study shows the joint effect of within‐individual longitudinal modelling of cognitive and functional performance with incident MCI/dementia. We show that modeling the baseline value only in analyses underestimates the association between cognition or function and risk of incident cognitive impairment. We conclude that longitudinal monitoring and assessment of cognition and function may result in more accurate estimates for risk. Future studies will pursue joint models that include fluid and imaging biomarkers.

